# A global view of T cell metabolism in systemic lupus erythematosus

**DOI:** 10.3389/fimmu.2024.1371708

**Published:** 2024-05-02

**Authors:** Andrew Goetz, Joy Cagmat, Maigan Brusko, Todd M. Brusko, Anna Rushin, Matthew Merritt, Timothy Garrett, Laurence Morel, Purushottam Dixit

**Affiliations:** ^1^ Department of Biomedical Engineering, Yale University, New Haven, CT, United States; ^2^ Department of Pathology, University of Florida, Gainesville, FL, United States; ^3^ Department of Biochemistry and Molecular Biology, University of Florida, Gainesville, FL, United States; ^4^ Department of Microbiology, Immunology & Molecular Genetics, University of Texas (UT) Health San Antonio, TX, United States; ^5^ Systems Biology Institute, Yale University, West Haven, CT, United States

**Keywords:** flux balance analysis, CD4 T cell, metabolic network, lupus, multiomic analyses

## Abstract

Impaired metabolism is recognized as an important contributor to pathogenicity of T cells in *Systemic Lupus Erythematosus* (SLE). Over the last two decades, we have acquired significant knowledge about the signaling and transcriptomic programs related to metabolic rewiring in healthy and SLE T cells. However, our understanding of metabolic network activity derives largely from studying metabolic pathways in isolation. Here, we argue that enzymatic activities are necessarily coupled through mass and energy balance constraints with in-built network-wide dependencies and compensation mechanisms. Therefore, metabolic rewiring of T cells in SLE must be understood in the context of the entire network, including changes in metabolic demands such as shifts in biomass composition and cytokine secretion rates as well as changes in uptake/excretion rates of multiple nutrients and waste products. As a way forward, we suggest cell physiology experiments and integration of orthogonal metabolic measurements through computational modeling towards a comprehensive understanding of T cell metabolism in lupus.

## Introduction

CD4^+^ T cells are an integral component of the adaptive immune system whose central function is clonal expansion and development of effector functions such as cytokine secretion and expression of co-stimulatory factors following exposure to antigens. To facilitate rapid proliferation, the metabolic network of T cells undergo a switch from a quiescent metabolic state characterized primarily by catabolic and homeostatic activities to a proliferative state characterized by anabolic activities (1).

The signaling program as well as the associated changes in gene expression that affect the metabolic switch during T cell proliferation have been thoroughly explored and reviewed elsewhere (2, 3). In contrast, our understanding of the changes in the metabolic state of cells is nascent but ever expanding (4). The first concrete explorations of metabolic rewiring accompanying the proliferation of healthy T cells occurred only two decades ago when it was shown that a switch to the proliferative state is accompanied by a significant upregulation of glucose uptake (5). This initial observation led to a flurry of research towards understanding the metabolic underpinnings of T cell function.

There is emergent consensus that impaired metabolic rewiring of T cells during proliferation is an essential feature of pathogenesis in several autoimmune disorders including systemic lupus erythematosus (SLE) (6), an autoimmune disorder that disproportionately affects women of Hispanic, African, and Asian ancestry (7). Importantly, these differences in metabolic rewiring have led to the development of several candidate therapeutics, some of which are currently being tested clinical trials, that target biochemical mechanisms that are orthogonal (8–10) to the standard of care for SLE based on immunosuppressants. Therefore, it is crucial that we gain a comprehensive understanding of impaired metabolic rewiring in SLE.

Most previous inquiries of SLE metabolism have studied metabolic pathways and nutrients in isolation, either by using only one approach (e.g. metabolomics or transcriptomics) or one metabolic pathway (e.g. glycolysis) as previously reviewed (11–13). In this perspective, we argue that the mammalian cellular metabolic network simultaneously carries out hundreds of interdependent chemical conversions (14), with large-scale dependencies and compensation mechanisms. Moreover, the metabolic network can be probed using multiple approaches, e.g. transcriptomics, metabolomics, and proteomics. Therefore, a comprehensive understanding of the impaired metabolic rewiring requires a simultaneous analysis of the exchange of nutrients and waste products and their relationship with cell proliferation and the cellular metabolic state.

Here, we first review metabolic rewiring in healthy T cells, followed by highlights of impaired rewiring in SLE. Next, we discuss how biophysical demands and constraints induce correlation across multiple pathways in the metabolic network and gaps in our knowledge. Finally, we sketch how biophysical measurements and computational integration of orthogonal metabolic, physiological, and transcriptomic data can estimate the metabolic state of T cells.

## Metabolic rewiring of healthy T cells

Metabolism of quiescent T cells is driven by homeostatic activities and is largely catabolic, requiring limited uptake of glucose, glutamine, and fatty acids, which are then routed through the oxidative pathways - oxidative phosphorylation (OXPHOS) and fatty acid oxidation (FAO) - to generate energy in the form of adenosine triphosphate (ATP) in the mitochondria. Consequently, quiescent T cells show very little aerobic glycolysis and low levels of lactate production (1). In contrast, metabolism of proliferating T cells is more active as it serves homeostatic, biosynthetic, and secretory functions. These functions demand a significantly higher energy requirement, utilized for polymerization of macromolecules as well as production of biomass precursors (amino acids, lipids, nucleotides etc.) from raw materials such as glucose, glutamine, and other amino acids.

Upon activation, T cells upregulate glucose consumption through membrane expression of glucose transporter 1 (GLUT1) (5). Most of the consumed glucose enters glycolysis where it produces NADH, ATP, and pyruvate. Additionally, glucose enters branched pathways including the pentose phosphate pathway (PPP), where it regenerates the cofactor NADPH and produces ribose-5-phosphate (R5P) (1, 15). NADPH is required for *de novo* synthesis of lipids and as a reducing equivalent in the regeneration of glutathione, a protective molecule that controls levels of reactive oxygen species (ROS). Glucose-derived R5P is used as the sugar backbone for nucleotide synthesis. A large fraction of pyruvate is excreted in the extracellular medium as lactate, in a phenomenon known as the Warburg effect (1, 16). The rest of the pyruvate enters the tricarboxylic acid (TCA) cycle, where it is used in the regeneration of ATP and in the synthesis of biomass precursors.

Proliferating T cells also uptake large amounts of glutamine (17). Consumed glutamine is used for the synthesis of proteins and nucleotides. Additionally, glutamine is converted to glutamate and then to the TCA cycle intermediate α -keto glutarate in a process called *glutaminolysis*. Glutamate, which is essential for epigenetic regulation of T cell differentiation (18, 19), is also used in the synthesis of nucleotides and glutathione. Recent work has also shown that surprisingly, supplementation of nonessential amino acids is crucial in T cell proliferation. T cells may be auxotrophic to alanine (20), which can in principle be synthesized from pyruvate in a single step using alanine transaminase. Similarly, serine which can be synthesized from glycolysis intermediate 3-phosphoglycerate, also needs to be supplemented externally in proliferating T cells (21). Consumed serine is used in the synthesis of proteins, lipid headgroups, nucleotides, and amino acids glycine and proline. Serine is also a key component of the one-carbon cycle which is essential for generating methyl groups that are used for DNA methylation.

## Pathogenic rewiring of T cells in SLE

There is now a growing consensus that impaired metabolic rewiring of T cells is central to the pathogenesis of SLE. It could be argued that the most well-documented metabolic impairment in SLE T cells, especially in human patients, are differences in mitochondrial utilization of glucose. When stimulated, SLE T cells show a marked increase in glycolysis and OXPHOS (10) with potentially lowered NADPH production through the PPP (22). SLE T cells are characterized by a high oxidative state and depleted levels of glutathione (23). Higher glycolysis in SLE T cells is achieved through a higher expression of GLUT1 (24) and higher OXPHOS (25) is achieved through an increased mitochondrial biomass (26). Paradoxically, mitochondria in SLE produce less ATP compared to healthy controls (HC) even though they are hyperpolarized (26). Additionally, evidence suggests that there are significant differences in glutamine (27) and lipid metabolism (28) in SLE T cells. A subset of CD4^+^ T cells producing IL-17 (Th17 cells) is expanded in SLE patients. Based on studies in mice, differentiation of Th17 cells relies strongly on glutaminolysis (29), as well as *de novo* lipid and cholesterol synthesis (30).

These key differences in metabolic rewiring have led to the identification of several potential therapeutic targets, some of which are currently being tested in clinical trials, that are orthogonal to the standard of care for SLE based on steroidal and nonsteroidal immunosuppressants (31–33). These include a combination therapy of 2-deoxy-D-glucose (2DG) and metformin that inhibit the first step of glycolysis and mitochondrial activity, respectively (9, 10), and inhibition of glutaminase, the first enzyme in glutaminolysis (34), in lupus-prone mice. Supplementation with N-acetyl cysteine, a reducing agent that is a precursor of cysteine, an amino acid used in glutathione synthesis, and treatment with mTOR inhibitor sirolimus or with metformin, have also shown promising results in SLE patients (35–38). The overarching goal to use cellular metabolism to selectively dampen the inflammatory autoreactive immune cells in SLE mirrors a growing effort to activate exhausted immune cells in the tumor microenvironment also through metabolic reprogramming (39).

## A need for system-wide study of metabolic changes

Several genetic, signaling, and metabolic investigations suggest that there are large-scale differences in the metabolism of HC and SLE T cells (13). However, most previous works, including those cited above, have studied metabolic pathways and nutrients in isolation, often using only one approach (e.g. metabolomics or transcriptomics) or one metabolic pathway (e.g. glycolysis). Importantly, however, the human metabolic network simultaneously carries out thousands of interdependent chemical conversions (40), with in-built large-scale dependencies and compensation mechanisms. Moreover, metabolic reactions are governed by tight constraints imposed by mass (14) and energy balance (41) as well as the laws of thermodynamics (41). Therefore, the exchange of nutrients and waste products and their relationship with cell proliferation and the cellular metabolic state must be understood simultaneously.

Such analysis requires quantitative knowledge of metabolic demands of proliferating T cells, both healthy as well as those in SLE patients. Unfortunately, even the most basic quantification of differences in metabolic demands is not available. For example, it is well established that SLE T cells have higher mitochondrial mass (26). But systematic changes in organelle distribution and their effect on overall biomass composition in SLE are not known. Similarly, the cytokine secretion profile is significantly altered between SLE and healthy T cells (42), with an increased production of pro-inflammatory cytokines in SLE. However, the metabolic burden of increased cytokine production by SLE T cells has not been quantified. Altered metabolic demands related to biomass and cytokine production have a direct effect on the nutrient uptake profile, downstream nutrient usage, and consequently the entire metabolic network. For example, increased protein (cytokine) production requires higher levels of synthesis of amino acids and higher ATP demand (43). Similarly, increased organelle mass requires higher *de novo* lipid synthesis, which in turn requires increased NADPH and NAD+ (44) regeneration rates.

Therefore, to elucidate metabolic driving mechanism of SLE pathogenesis and to discover new druggable targets for SLE and other autoimmune disorders, we need a systematic and unbiased characterization of the differences in metabolic requirements as well as metabolic network activity of SLE and healthy T cells.

## Towards a system-wide understanding of T cell metabolism using computational integration

How do we obtain a network-wide characterization of T cell metabolic activity? Advances in genomics, proteomics, and metabolomics allow us to obtain a high dimensional and high-resolution characterization of cellular metabolism. However, these measurements only provide indirect information about the *metabolic state* of cells - network-scale enzyme activity or reaction rates. This is because reaction fluxes are a complex function of enzyme kinetics (45), thermodynamics (45), metabolomics (45), and gene expression (46), and therefore are not uniquely determined by -*omics* characterizations. For example, metabolite levels may be high either because of a high rate of production or a low rate of clearance. Similarly, high gene expression levels may imply higher reaction rates or a compensatory mechanism to maintain constant reaction rates. Therefore, typical *omics* measurements cannot be directly used to infer differences in metabolic states of cells. Moreover, while labeled carbon experiments allow estimation of intracellular fluxes in bacteria (47, 48), a direct measurement of most intracellular fluxes is not possible in mammalian cells, owing to compartmentalization (47). Consequently, metabolic states cannot be characterized using direct measurements either.

**Figure 1:**
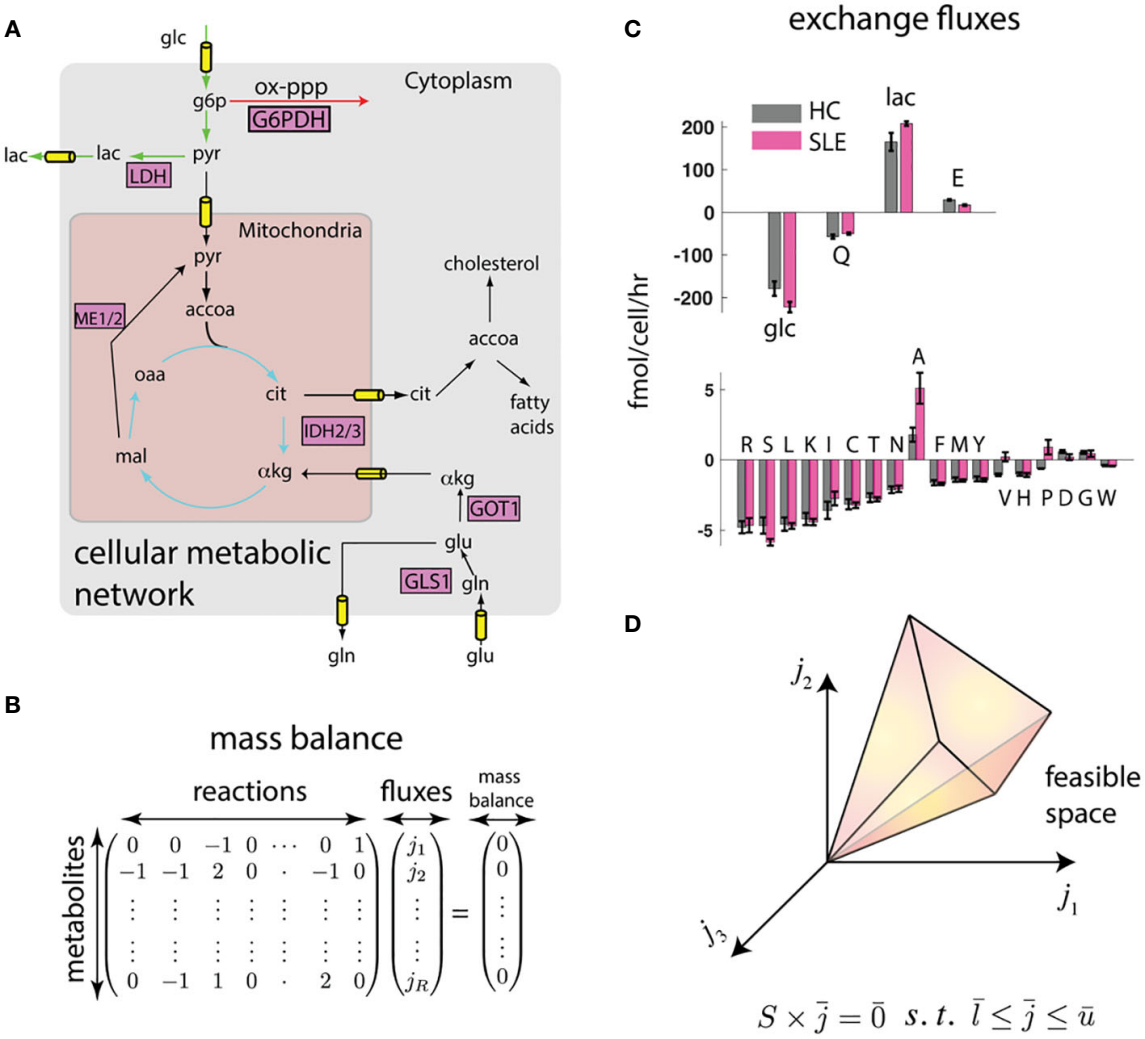
Flux balance (FBA) framework integrates biophysical constraints and measurements with transcriptomics. **(A)** Genome-scale map of metabolic interconversions (40) is expressed in **(B)** as the stoichiometric matrix S whose entries $S_{mr}$ denote the participation of metabolites m in reactions r . If metabolite concentrations are at steady state, the vector $\bar{j}$ of reaction rates must be in the null space of S . **(C)** Constraints to this metabolic map can be provided in the form of consumption and release (CORE) rates. We show measured CORE rates of high flux metabolites (glucose (glc), glutamine (Q), lactate (lac), and glutamate (E)) and low flux nutrients (other amino acids) separately. Error bars represent standard error of the mean from measurements on T cells derived from n = 4 mice each (see Methods). **(D)** These linear constraints and reasonable upper and lower bounds on reaction fluxes ( $\bar{l}\leq \bar{j}\leq \bar{u}$ ) defines a feasible space. The feasible space can be further constrained by biophysical and transcriptomic measurements.

A way to overcome these limitations is through computational modeling and data integration. Notably, *omics*-based indirect characterizations can be integrated with cell physiological information such as proliferation rate, cell size, cytokine excretion rate, and crucially, the consumption and release rates (CORE) of several nutrients and waste products, which can be accurately measured in cell culture using mass spectrometry (49). This integration can be achieved using the flux balance analysis (FBA) framework (14) (**Figure 1**).

While *omics* data do not uniquely determine the fluxes using FBA, they do constrain the plausible fluxes to a *feasible space (* 14 *)* (**Figure 1D**). To further identify unique fluxes, FBA approaches typically invoke optimality of an underlying objective function, for example, fast growth or maximum yield, to obtain a unique flux solution (50). While these optimality-based approaches have been quite successful in modeling the metabolism of single cell organisms (50), mammalian cells have not necessarily evolved for fast growth, and the specific metabolic objective (e.g., lipid production, cytokine secretion, cell proliferation) may depend on cell type and extracellular environment, and may not even be metabolic in nature.

In the absence of direct measurements, a conceptually straightforward way to obtain an estimate of intracellular fluxes is probabilistic sampling of the feasible space using a Bayesian framework and Markov Chain Monte Carlo (51) that integrates all available measurements and biophysical constraints. For example, the feasible space defined by CORE measurements and proliferation rates can be further constrained using transcriptomics by requiring fluxes to align with reaction activity scores (46). Moreover, fluxes can be required to satisfy energy balance (41), thereby eliminating unrealistic loops that satisfy mass balance but violate the second law of thermodynamics.

## Computational integration predicts network-wide changes in metabolic activity

To test whether such an integration can consistently identify potential differences in metabolic states of SLE and healthy T cells, we performed preliminary analyses on splenic CD4^+^ T cells from lupus-prone (TC) and healthy control (B6) mice (see methods). In this proof of principle work, CD4^+^ T cells that do not express surface markers associated with receptor activation (i.e. “naïve”) were used to eliminate the differences in activation status that exist between TC and B6 T cells. To obtain constrained metabolic rates, we measured uptake and excretion rates of amino acids, glucose, and lactate in these T cells that were activated *in vitro* through their CD3ϵ, a signaling subunit of the T cell receptor, and the co-receptor CD28. As shown in **Figure 1C**, there are large scale differences in nutrient exchange profile with highest exchange fluxes were glucose/lactate and glutamine/glutamate. Surprisingly, while most amino acids were consumed, glutamate, alanine, aspartate, and glycine were excreted. Importantly, there were significant differences in exchange fluxes between T cells from TC and B6 mice. These data already hint at global metabolic differences between TC and B6 T cells that could not be detected by traditional metabolomic analyses.

**Figure 2:**
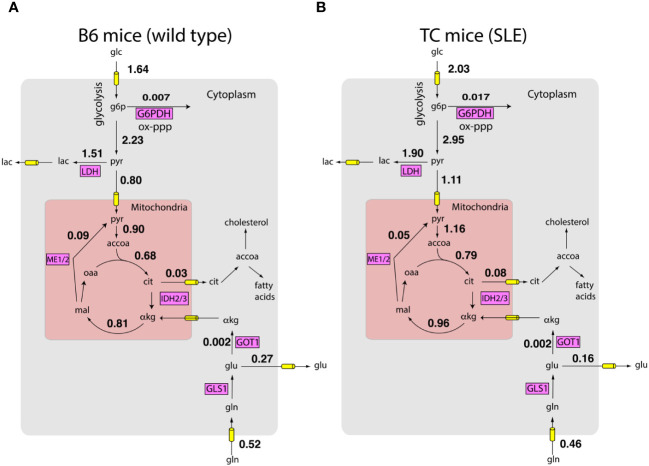
Inferred metabolic activity HC and SLE T cells. Inferred intracellular metabolic fluxes in *in vitro* stimulated CD4^+^ T cells from HC **(A)** and SLE **(B)** mice. Shown are key metabolic reactions in glycolysis, TCA cycle, and oxidative pentose phosphate pathway. Key enzymes in these pathways are shown in magenta boxes. Numbers represent model estimated reaction rates in millimoles per gram dry cell weight per hour (mmol/g-DW-hr). The fluxes are reported as an average of measurements on T cells harvested from n = 4 mice for each condition.

Next, we integrated the measured the uptake and excretion rates with measured gene expression of metabolic enzymes using a model of the human metabolic network and the FBA framework. By sampling the feasible space of intracellular fluxes constructed using the FBA framework (see methods), we obtained posterior distributions of intracellular fluxes. Fluxes from these distributions satisfied mass balance and laws of thermodynamics as imposed by the metabolic network. The fluxes were constrained to reproduce the measured growth rate and nutrient exchange rates. The fluxes were also biased to align with gene expression profiles obtained by RNA sequencing. **Figures 2A, B** show that according to the Bayesian model, there are network-wide differences in the metabolism of T cells. Overall, TC T cells had a more active metabolism, ∼76% of the reactions in the model had a higher flux in the lupus mice. These preliminary analyses show that simple biophysical and metabolic experiments, combined with transcriptomics and genome-wide metabolic models can allow us to estimate a detailed picture of intracellular fluxes.

These predictions offer a systematic route to integrate measured information and thereby obtain testable hypotheses concerning the role of shifts in metabolic activity in SLE pathogenesis. For example, consistent with known SLE pathology, glucose consumption by TC mice is significantly higher compared to B6 mice (10). Surprisingly, while lactate excretion by TC mice is also higher (10, 13), the fraction of pyruvate excreted as lactate is similar between B6 and TC mice ( 67±6.5% vs 65±5% , Wilcoxon rank sum test p=0.49 , *n* = 4 mice for each condition). This suggests that higher lactate production by T cells of TC mice may largely be explained by their higher proliferation rate and consequent higher metabolic activity. Indeed, consistent with the model prediction, despite shifts in glucose and lactate utilization, the ratio of oxygen consumption rate (OCR) to extracellular acidification rate (ECAR), a proxy for pyruvate utilization, is similar between B6 and TC mice (10). In contrast, the amount of glucose entering the oxidative pentose phosphate pathway is higher in T cells from TC mice compared to B6 mice ( 0.017±0.01 vs 0.07±0.004 , Wilcoxon rank sum test p=0.0571 , n=4 mice for each condition). This may reflect not only the higher demand for anabolic NADPH but also the NADPH required to regenerate glutathione. While fluxes in **Figure 2** are only predictions from the computational model, these examples show that they offer numerous testable hypotheses about the utilization of nutrients.

These preliminary analyses show that the integration of biophysical measurements, gene expression data, and estimates of consumption and release rates of metabolites can be accomplished using a unifying Bayesian framework to obtain unbiased predictions about the metabolic states of cells.

## Outlook

Targeting the changes in the metabolic network is an attractive therapeutic avenue in treating autoimmune disorders like SLE that is orthogonal to current immunosuppressant-based treatments or even more novel biologics targeting specific immunological cells or pathways. However, our current understanding of metabolic differences in SLE and healthy T cells is limited to studies performed on individual pathways in isolation. While it remains experimentally challenging to directly probe the entire metabolic network, computational methods can integrate several pieces of multi-omics information and biophysical constraints to predict network activity. We believe that these approaches will serve as an important tool in forming global understanding of T cell metabolism in health and disease.

## Methods

### Mice and cell culture preparation

B6.Sle1.Sle2.Sle3 (TC) mice have been previously described (52). C57BL6/J (B6) mice were originally purchased from the Jackson Laboratory. Both strains were maintained at the University of Florida. Naïve CD4^+^ T cells were purified by negative selection with antibody-coated magnetic beads (Miltenyi Biotech) from the spleen of n = 4 B6 and TC mice and then stimulated with plate-bound anti-CD3 (145-2C11, 2 μg/ml) and soluble anti-CD28 (37.51, 1 μg/ml) antibodies, both purchased from BD Biosciences, in serum-free RPMI medium as previously described (10) with the addition of 25 mM glucose and 10 mM glutamine. Naïve CD4^+^ T cells were used instead of total CD4^+^ T cells to limit differences due to an increased frequency of activated T cells in lupus. Cell numbers were measured in stimulated cells on day 2 and day 3 of growth to fit an exponential growth parameter.

### Measurement of consumption and release rates

Consumption and release (exchange) rates of amino acids were measured using mass spectrometric analysis of cell culture supernatant using LC/MS/MS as described previously (49) using an amino acid kit from Kairos Waters with a ThermoScientific TSQ Altis with Vanquish UHPLC for all mass spectrometry analyses. Glucose and lactate concentrations were measured using NMR. Briefly, absolute concentrations of metabolites were measured in the supernatant on day 2 and 3 and exchange rates were estimated by dividing the net change in metabolite concentration by the area under the curve of cell number profile ^1^H NMR spectra were acquired using a 14 T magnet equipped with a 5 mm TXI CryoProbe (Bruker BioSpin). Spectra were collected using a spectral width of 12 (32768 complex data points) and an acquisition time of 2.27 s using the NOESYPR1D experiment. Data were processed using MestReNova (v14.2.1). Glucose and lactate concentrations in media were calculated from an internal standard of 0.5 mM DSS (Sigma-Millipore) and 0.5 mM pyrazine (Sigma-Millipore) using appropriate T1 corrections for the standards versus the metabolites.

### Gene expression using single cell RNA sequencing

Magnetic bead isolated cell specimens were confirmed to have sufficient viability (>85%) with Acridine Orange/Propidium iodide staining and a Nexcelom Cellometer, then prepared for droplet encapsulation using the 3’ Single Cell Profiling reagent kit (version 3.1) (10x Genomics) and manufacturer’s instructions for cDNA library preparation. Libraries were sequenced on a NovaSeq S4 flow cell. Demultiplexed libraries were analyzed using Cellranger.

### Computational algorithm to predict intracellular fluxes

Exchange fluxes and growth rates were used in a flux balance model of the human metabolic network. The metabolic model comprises the union of all reactions that enzymes coded in the human genome can support. To obtain a tissue specific pruned model, we used the measured exchange rates and proliferation rates as described before (44). The constraints imposed by steady state metabolite concentrations, exchange rates, and proliferation rates described a convex polytope of plausible intracellular fluxes. We sampled intracellular fluxes from this polytope using rejection sampling that rejected flux distributions that violated the second law of thermodynamics (41). Additionally, we biased the flux distribution using gene expression that was converted into reaction activity scores (46, 53) - aggregate expression levels of genes that correspond to a given metabolic reaction. The data and the scripts used for this preliminary analysis can be found at https://github.com/adgoetz186/Flux_Code.

## Data availability statement

The original contributions presented in the study are included in the article/supplementary materials. Further inquiries can be directed to the corresponding authors.

## Ethics statement

The animal study was approved by Institutional Review Board of the University of Florida. The study was conducted in accordance with the local legislation and institutional requirements.

## Author contributions

AG: Conceptualization, Data curation, Formal analysis, Methodology, Visualization, Investigation, Writing – review & editing. LM: Funding acquisition, Methodology, Writing – review & editing, Conceptualization, Investigation, Resources. PD: Conceptualization, Funding acquisition, Visualization, Writing – original draft, Writing – review & editing, Supervision. JC: Writing – review & editing, Data curation, Methodology, Resources. MB: Writing – review & editing, Data curation, Methodology, Resources. TB: Writing – review & editing, Data curation, Supervision, Resources. AR: Writing – review & editing, Data curation, Methodology, Resources. MM: Writing – review & editing, Supervision, Resources. TG: Data curation, Supervision, Resources, Writing – review & editing.
